# Gastrocnemius electrical stimulation increases ankle dorsiflexion strength in patients with post-acute sequelae of SARS-COV-2 (PASC): a double-blind randomized controlled trial

**DOI:** 10.1038/s41598-024-68100-8

**Published:** 2024-08-02

**Authors:** Myeounggon Lee, Alejandro Zulbaran-Rojas, Miguel Bargas-Ochoa, Bernardo Martinez-Leal, Rasha Bara, Areli Flores-Camargo, M. G. Finco, Ram kinker Mishra, Jaewon Beom, Dipaben Modi, Fidaa Shaib, Bijan Najafi

**Affiliations:** 1 Digital Health Access Center (DiHAC), Division of Vascular Surgery and Endovascular Therapy, Michael E. DeBakey Department of Surgery, Baylor College of Medicine, 7200 Cambridge St, B01.529, Houston, TX 77030 USA; 2 Department of Physical Therapy at the University of North Texas Health Science Center in Fort Worth, Fort Worth, TX USA; 3 H. Ben Taub Department of Physical Medicine and Rehabilitation, Baylor College of Medicine, Houston, TX USA; 4 Department of Rehabilitation Medicine, Seoul National University College of Medicine, Seoul National University Bundang Hospital, Seongnam, Korea; 5 Department of Pulmonary Critical Care, Baylor College of Medicine, Houston, TX USA

**Keywords:** Rehabilitation, Biomedical engineering

## Abstract

Post-Acute sequelae of SARS-CoV-2 (PASC) is a multisystem disorder causing persistent musculoskeletal deconditioning and reduced lower extremity strength. Electrical stimulation (E-Stim) to the gastrocnemius muscle can enhance strength outcomes by increasing the frequency of muscle fiber activation. We investigated its effect on individuals with PASC. Participants were randomized into intervention (IG) or control (CG) groups. The IG self-administered daily one-hour E-Stim to both their gastrocnemius muscles using a functional device over 4-week, while the CG used a sham device. Primary outcomes were ankle dorsiflexion strength assessed via dynamometry during maximum voluntary contractions, and gastrocnemius voluntary activation (GVA) via surface electromyography. The secondary outcome assessed activities of daily living (ADL), instrumental ADL, and mobility queries. Percentage improvement was calculated. Eighteen patients were analyzed (IG = 10; CG = 8). After 4 week, the IG showed a significantly higher improvement in ankle dorsiflexion strength (222.64%) compared to the CG (51.27%, *p* = 0.002). Additionally, the IG’s ankle dorsiflexion strength improvement significantly correlated with GVA improvement (rho = 0.782) at 4 week. The secondary outcomes did not reveal significant changes in neither group. Self-administered gastrocnemius E-Stim improves ankle dorsiflexion strength in individuals with PASC. However, larger sample sizes and longer interventions are needed to validate these findings.

## Introduction

Post-acute sequelae of Sars-CoV-2 (PASC) is a multisystem condition characterized for persistent symptoms in different organs following COVID-19 clearance^1^. One of the most affected systems by PASC is the musculoskeletal, encompassing up to 41% of patients reporting muscle weakness and fatigue as early as hospital discharge and may last for years^2,3^. Particularly, PASC is more prominent in the lower extremities (LE) of those who were hospitalized or needed prolonged bed rest^4^.

Various hypothesized pathways by which the musculoskeletal system is affected by Sars-CoV-2 have been proposed^5^. Either by direct myocyte invasion through ACE2 receptors, or by muscle wasting during acute COVID-19 infection suggested by present indicators (i.e., elevated creatinine kinase, rhabdomyolysis)^6,7^. Whether these hypothesized mechanisms are true, reduced physical performance and ability to carry out daily activities has been associated with musculoskeletal PASC^8–10^.

Exercise and increased physical activity are known to improve musculoskeletal strength in people with muscle deconditioning due to prolonged hospitalization^11^. However, in individuals with PASC with previous hospitalization, physical activity must be carefully monitored as studies have shown this population can present silent hypoxia and exercise intolerance when performing moderate to rigorous exercise^12^.

Electrical stimulation (E-Stim) therapy has shown to improve musculoskeletal function in older adults with low physical activity, and this modality produces a similar response to exercise in people with sarcopenia who are unable to engage in normal physical activity^13^. When delivered to the lower leg muscle groups (gastrocnemius and soleus), E-Stim has shown to improve endurance and strength in critically ill COVID-19 patients^4^. The mechanism of action is based on activation of ankle joint muscles, which play an important role in fundamental movements such as gait and balance^14^, that aid to execute simple tasks such as activities of daily living^15^. Particularly, individuals with PASC have shown lack of independence in these activities^16–18^; thus, safe rehabilitation methods are warrant to support their reintegration to pre-COVID mobility conditions.

In individuals with musculoskeletal sequelae due to hospitalization, E-Stim has shown to improve LE muscle strength^19,20^. However, this outcome has not been explored in individuals with PASC and musculoskeletal sequelae. In this study, we examined the potential of daily, self-administered, home-based gastrocnemius E-Stim to aid in the recovery of ankle strength and muscle activation in this population. Our hypotheses are: (1) a 4 week E-Stim therapy will improve the ankle dorsiflexion strength and gastrocnemius activation of participants, and (2) the ankle dorsiflexion strength magnitude of improvement will correlate with the gastrocnemius activation magnitude of improvement at the end of the study.

## Methods

### Study design

This is a secondary analysis of a double-blinded randomized controlled trial in patients presenting persistent LE musculoskeletal deconditioning symptoms (i.e., weakness, atrophy, numbness, and/or pain) for at least 3 months after clearance of acute COVID-19 infection that were not present before. The details regarding clinical trial design, inclusion and exclusion criteria, recruitment strategy, and results focusing on muscle endurance and perfusion have been previously published^21^. Participants were recruited from Baylor College of Medicine’s (BCM) Post-COVID Care Clinic (Houston, TX, USA), or self-referred by contacting our research staff through the ClinicalTrials.gov website information from November 2021 to May 2022, after readings and signing an informed consent form approved by the BCM Institutional Review Board (IRB number: H-47781). The protocol was registered in ClinicalTrials.gov (Identifier: NCT05198466, 01/20/2022). The methods used were in accordance with the relevant guidelines and regulations, and the Helsinki Declaration.

### Grouping and electrical stimulation intervention

**Figure 1:**
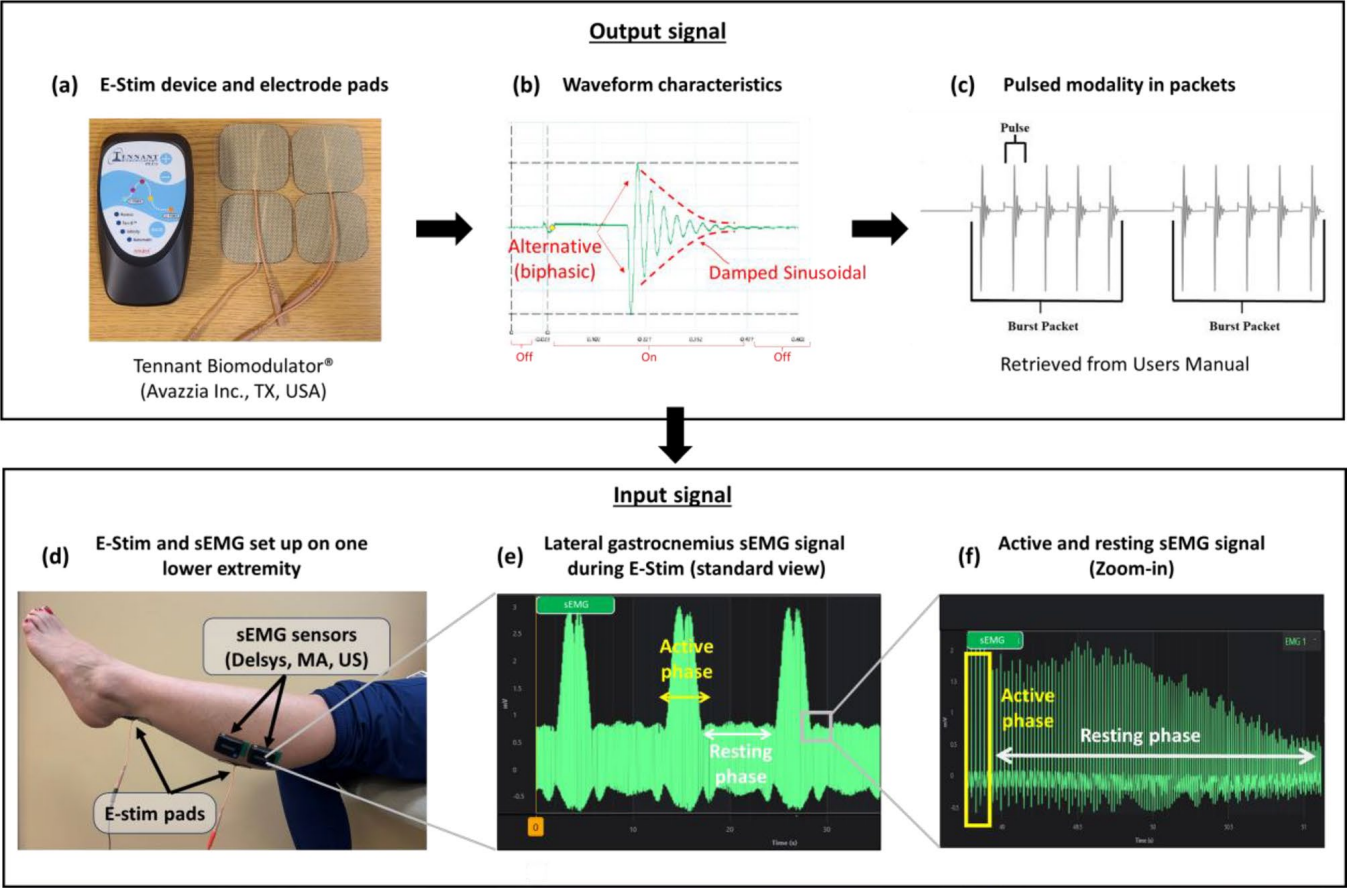
Experimental set-up: (**a**) is the E-Stim device; (**b**) is the waveform characteristics providing the E-Stim device; (**c**) is the pulsed modality in packets from the E-Sti, device; (**d**) is describing the locations of the sEMG sensors and E-Stim pads; (**e**) is the lateral gastrocnemius sEMG signal during the E-Stim; and (**f**) is describing the active and resting sEMG signal during the E-Stim.

The intervention protocol has been previously described^21^. Briefly, participants were randomized into intervention group (IG) and control group (CG) at a 1:1 ratio using a computer-generated list, followed by sequential allocation. Participants and their caregivers were unaware of their group allocation. Over the course of 4 weeks, the IG received daily one-hour E-Stim to the gastrocnemius via four-electrode adhesive pads (two placed on each leg) connected to a four-pin lead wire coming from a wearable E-Stim device (Tennant Biomodulator®, Avazzia Inc., Dallas, TX, USA, Fig. 1a). One electrode pad was attached between the proximal and medial gastrocnemius in a strong triggering point^22^. The other electrode pad was attached over the proximal Achilles tendon (Fig. 1d). This strategic placement was designed to simultaneously stimulate the medial and lateral gastrocnemius along with the soleus muscles^23,24^. The CG used an identical sham device for the same period. The total therapy sessions were between 28–30 (1 h per day around 4 weeks).

### Electrical stimulation current characteristics

E-Stim was administered using an interactive high voltage pulsed alternative current (HVPAC) manifesting as an asymmetrical damped sinusoidal waveform (Fig. 1b)^25^. The electrical properties of the waveform alter in response to varying tissue characteristics, such as skin impedance, until a stable conductivity is achieved, marking the formation of a closed-loop E-Stim system^25,26^.

The HVPAC mode was configured at a power level of 50, resulting in an intensity of − 0.55 milliamperes (mA). Electrical pulses ranged from 0.3 to 1.3 ms (ms) and were grouped into packets of 5 pulses, each separated by 1.62 ms (Fig. 1c). These packets were delivered at a frequency of 21 to 129 Hertz (packets per second). Each packet lasted 7.2 ms, with a rest phase between packets ranging from 8 to 33 ms.

The HVPAC output signal placed in the gastrocnemius was recorded via sEMG. The resulting electromyogram reading displayed a cyclic pattern of electrical conductivity within deep and superficial tissues (Fig. 1e). This cycle alternated between a peak of high neuromuscular electrical conductivity, referred to as the active phase, lasting 3 s, followed by a decline to a flat phase or resting phase, where minimal neuromuscular electrical conductivity lasts for 6 s (Fig. 1f). This cycle was repeated every minute, resulting in 24 s of muscle contraction × 36 s of muscle relaxation, thus, 24 min of muscle contraction × 36 min of muscle relaxation per hour. The recurring cycle continued throughout the duration of the stimulation and did not elicit tetanic contraction as confirmed by simple view and sEMG reading.

The output and input currents were identical and constant in all E-Stim sessions. The setting was predetermined and standardized in all the devices. This set-up was reported to be harmless in previous clinical trials for muscle deconditioning^4^. Of note, the device of the present study is FDA-cleared for pain relief^25^, thus, the therapeutic intervention (neuromuscular E-Stim) for this study was exploratory.

### Primary outcomes

All patients were assessed at baseline and 4-week follow-up. During each visit, all participants’ skin was cleaned with alcohol and prep gel (Nuprep, CO, US) to minimize impedance, and two surface electromyography sensors (sEMG, Delsys Trino Wireless EMG System, MA, US) were placed on the lateral gastrocnemius of each leg^27^ along with the E-Stim pads previously described (Fig. 1a).This sEMG location was modified from the Surface Electromyography for a Non-Invasive Assessment of Muscles (SENIAM) guidelines^27^, to avoid overlapping noise elicited from the E-Stim pads^27,28^ (Fig. 1a). Then, participants were sat in Fowler’s position with their heels hanging off the leg rest to measure ankle dorsiflexion isometric strength using a low-cost portable dynamometer (RoMech Digital Hanging Scale), known as a reliable tool for muscle strength assessment and validated in comparison to the gold standard isokinetic dynamometer (ICC: 0.993–0.995)^29^. To begin the assessment, the dynamometer was set to zero kilograms and participants performed three isometric dorsiflexions at maximum voluntary contraction (MVC) for 5 s and resting 30 s in-between on each foot. Then, averaged data from both feet was calculated. Simultaneously, the sEMG signal from each of the lateral gastrocnemius was recorded, defined as gastrocnemius voluntary activation (GVA)^4^. The average data from both GVA was calculated. Of note, the MVC assessment not only evaluates the agonist muscle’s activity but also the antagonist muscles’ activity, which are more pronounced during dorsiflexion rather than plantar flexion^30^. Thus, isometric dorsiflexion tests can provide a comprehensive evaluation of the antagonist muscles that provide strength to the ankle.

### Electromyography data analysis

The sEMG signal was collected at 2000 Hz and filtered using a 4th order Butterworth band-pass filter with cutoff frequencies of 20 and 350 Hz. To assess GVA, the filtered sEMG signal was full-wave rectified and smoothed using a moving average to estimate the sEMG linear envelope^31^.

To quantify GVA during MVC, integrated EMG (iEMG) was calculated^32^. The iEMG was normalized by the average iEMG value extracted during the trial to compare the iEMG values on different visits^33^. This method quantifies the amount of muscle fiber activation by motor units^34^, and an increase in iEMG indicates that more motor units are being recruited for muscle activation, which can be interpreted as an increase in muscular strength^32^. The iEMG values from two EMG sensors in each leg were averaged and reported. The EMG signal analysis was performed using custom-made software programmed in MATLAB (The MathWorks Inc., Natick, MA, USA).

### Secondary outcomes

Baseline clinical characteristics and demographics were obtained from the patients’ electronic medical records. On the baseline and 4 week assessment visits, participants were asked a series of questionnaires related to daily and physical activity, that included the Katz index of independence in Activities of Daily Living^35^, the Lawton-Brody Instrumental Activities of Daily Living (IADL)^36^, and mobility and tiredness^37^. These queries are crucial for evaluating independence and functional status. Difficulties in these activities indicate a lower quality of life and increased dependence on others^16,17^.

### Power analysis

A power analysis (G*power version 3.1.6) was carried out to determine the minimum sample size based on specific criteria. The sample size was estimated based on our previous study^4^, in which the effectiveness of E-Stim demonstrated a significant improvement in ankle dorsiflexion strength in COVID-19 patients. These included a moderate effect size (f = 0.385), 80% power, 5% alpha, 2 groups, 2 repeated measurements, and a 0.5 correlation between the repeated measurements. As a result, the study required 16 samples, but to account for a potential dropout rate of up to 10%, a total of 18 samples were needed to detect significant results.

### Statistical analysis

Data normality was evaluated using the Shapiro–Wilk test, accepting a *p* value greater than 0.05 as indicative of normal distribution. For comparing groups at baseline, we used independent t-tests for continuous variables and chi-square tests for categorical variables when the data followed a normal distribution. If this assumption was not met, the Mann–Whitney U tests were applied instead. To assess the interaction effect between the group (intervention vs. control) and the assessment (Baseline vs. 4-week), we employed Generalized Estimating Equations (GEE) adjusted to only one covariate as a conservative analysis for small sample sizes. This also allowed us to calculate the estimated mean and standard error values, as well as 95% of confidence interval (CI). The baseline outcomes (i.e., ankle dorsiflexion strength) that showed significant difference between groups were adjusted as covariates in the main analysis. A secondary analysis adjusting all significant differences between groups at baseline (i.e., BMI, ankle dorsiflexion strength) was described in the supplementary materials.

Additionally, we computed the percentage (%) improvement in GVA and ankle dorsiflexion strength using the formula: ([4 week—baseline]/[baseline]*100), considering the baseline value as 0% (i.e., [baseline − baseline]/[baseline]*100 = 0). The effect size was measured using Cohen’s d. A low effect size is less than 0.5, moderate is 0.5–0.79, and large is above 0.8^38^. To examine the correlation of GVA and ankle dorsiflexion strength at 4 week in each group, Spearman’s sign-rank correlation analysis was conducted. For assessing E-Stim effectiveness on the gastrocnemius muscle, we calculated the delta (∆) values (i.e., 4-week – baseline) for GVA and ankle dorsiflexion strength values, investigating associations between ∆GVA and ∆dorsi-flexion strength values improvement. All statistical analyses were performed using SPSS 29.0 (IBM, Chicago, IL, USA), and the was set at 0.05.

### Ethics approval

The studies involving human participants were reviewed and approved by the Institutional Review Board for Human Subject Research for Baylor College of Medicine and Affiliated Hospitals (BCM IRB: H-47781; Initial submit date: 05/13/2020). The patients/participants provided their written informed consent to participate in this study. The informed consent was obtained from all participants and/or their legal guardians. This study was carried out in accordance with the declaration of Helsinki.

## Results

**Figure 2:**
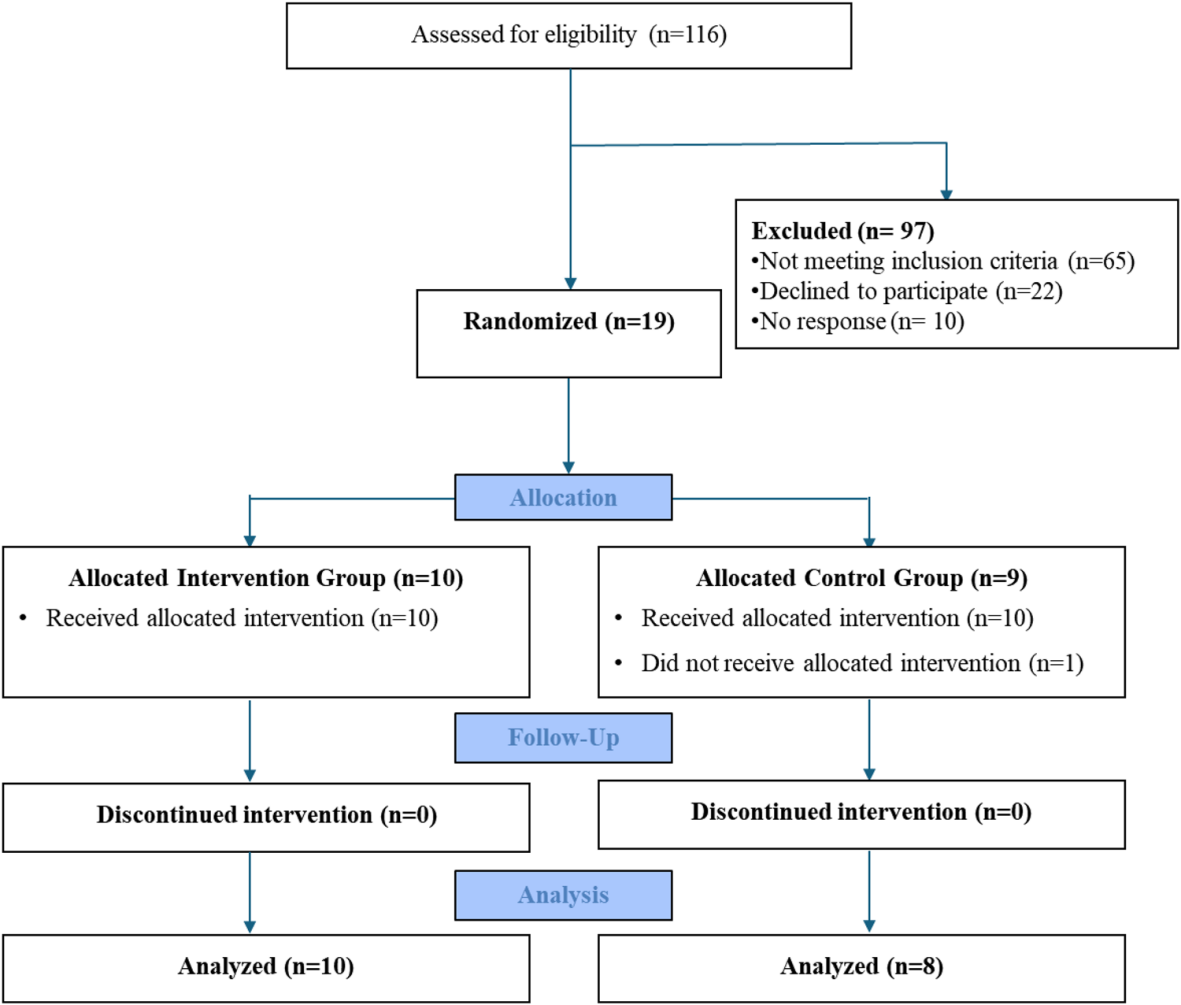
Consort flow diagram^21^.

This secondary analysis of our initial study^21^ included 116 potential candidates who were screened, however 65 did not meet the eligible criteria, 22 refused to participate, and 10 did not respond to phone calls. As a result, 19 participants were recruited; however, one participant in CG withdrew from the study due to non-compliance. In total, 18 participants successfully completed all assessments and interventions over a 4-week period. The CONSORT flow chart and baseline characteristics of this study’s population have been described in our initial analysis (Fig. 2). In summary, the IG showed lower BMI (*p* = 0.016, d = 1.280), higher pneumonia during COVID-19 acute infection (*p* = 0.043, d = 1.089), and higher oxygen supplementation at home (*p* = 0.040, d = 1.107) compared to the CG (Table 1). Ankle dorsiflexion strength at baseline assessment was significantly higher in the CG compared to the IG (*p* = 0.014, d = 0.901).

**Table 1:** Demographics and clinical characteristics.

	Intervention	Control	*p* value
	(n = 10)	(n = 8)	(Effect-size)
Demographics			
Age (years)	51.10 ± 9.86	52.38 ± 7.44	0.766 (0.144)
Sex: Female, n (%)	7 (70)	6 (75)	0.814 (0.111)
BMI (kg/m^2^)	30.28 ± 5.20	37.03 ± 5.35	0.016 (1.280)*
Ethnicity: Non-Hispanic, n (%)	7 (70)	7 (87.5)	0.570 (0.516)
Clinical characteristics			
Diabetes, n (%)	3 (30)	3 (37.5)	0.737 (0.158)
Hypertension, n (%)	5 (50)	3 (37.5)	0.596 (0.252)
Hyperlipidemia, n (%)	2 (20)	3 (37.5)	0.410 (0.079)
Pneumonia during COVID-19, n (%)	4 (40)	0	0.043 (1.089)*
Current Shortness of breath, n (%)	2 (20)	3 (37.5)	0.410 (0.395)
Under current respiratory rehabilitation, n (%)	2 (20)	3 (37.5)	0.410 (0.395)
Walking aid, n (%)	4 (40)	1 (12.5)	0.236 (0.875)
Days of hospitalization (days)	28.10 ± 27.82	7.75 ± 6.23	0.061 (0.957)
Supplemental oxygen during hospitalization, n (%)	7 (70)	5 (62.5)	0.737 (0.159)
ICU admission, n (%)	6 (60)	3 (37.5)	0.343 (0.459)
Persistency of symptoms after acute infection (days)	295.60 ± 224.92	304.50 ± 179.45	1.000 (0.000)
Oxygen use at home, n (%)	6 (60)	1 (12.5)	0.040 (1.107)*

Variables are expressed as means ± standard deviation. BMI: Body mass index. COVID-19: Coronavirus disease of 2019. ICU: Intensive care unit. The asterisk denotes a significant between-group difference (*p* < 0.05). This table references content from the paper by Zulbaran‐Rojas et al. (2023)^21^.

**Figure 3:**
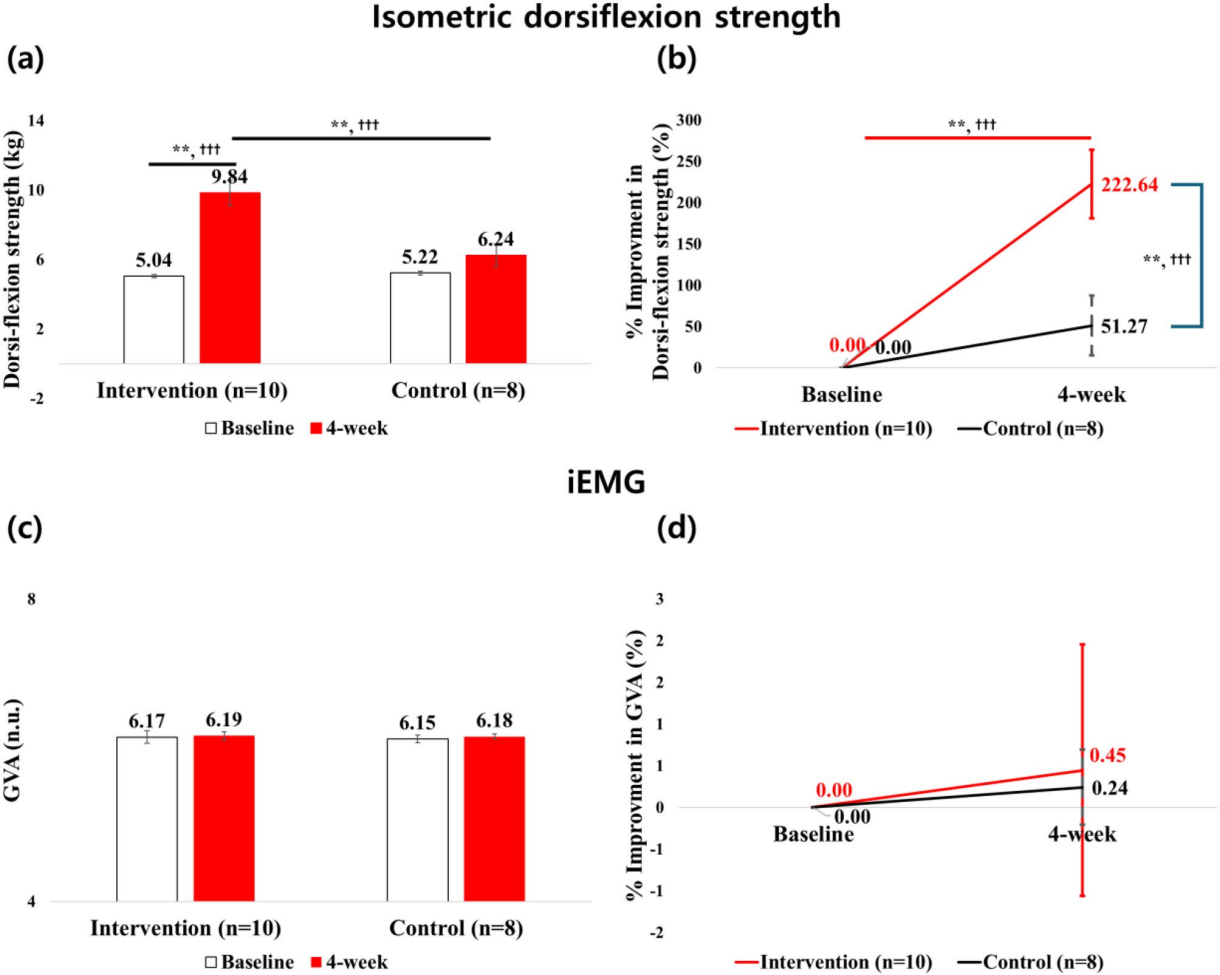
Ankle dorsiflexion strength and EMG for gastrocnemius between baseline and 4 week visits for absolute (i.e., figure in left side) and %improvement (i.e., figure in right side) values: (**a**) is the dorsiflexion strength and baseline ankle strength was adjusted as a covariance; (**b**) is the improvement in dorsiflexion strength; (**c**) is the EMG for gastrocnemius activation during MVC (GVA); and (**d**) is the improvement in GVA; * = *p* < 0.05, ** = *p* < 0.01; ^†^ = low effect-size, ^† †^ = moderate effect-size, ^† † †^ = strong effect-size.

At 4 week, the IG showed a significant improvement in ankle dorsiflexion strength compared to baseline (*p* < 0.001, d = 2.790; 95% CI at baseline: 4.85–5.23; and 4 week: 8.43–11.24), while no significant change occurred in the CG (*p* = 0.078, d = 0.943; 95% CI at baseline: 5.00–5.44; and 4 week: 4.94–7.55) (Fig. 3a). Additionally, a significant interaction effect for group × assessment in favor of the IG was seen for ankle dorsiflexion strength (*p* < 0.001, d = 1.225).

Percentage improvement in ankle dorsiflexion strength significantly increased at 4 week from baseline in the IG (*p* < 0.001, d = 1.336; 95% CI at 4 week: 141.31–303.97), while the CG did not improve (*p* = 0.153, d = 0.714; 95% CI at 4 week: − 19.06–121.59). Comparison between groups showed the IG had greater improvement than the CG (222.64% vs. 51.27%, *p* = 0.002, d = 1.439, Fig. 3b) at 4 week. Additionally, there was a significant interaction effect for group × assessment in favor of the IG for percentage improvement in ankle dorsiflexion strength (*p* = 0.002, d = 1.220).

There were no significant differences for GVA (*p* = 0.894, d = 0.032, Fig. 3c) as well as percentage improvement in GVA (*p* = 0.896, d = 0.044, Fig. 3d) for interaction effect for group × assessment.

**Figure 4:**
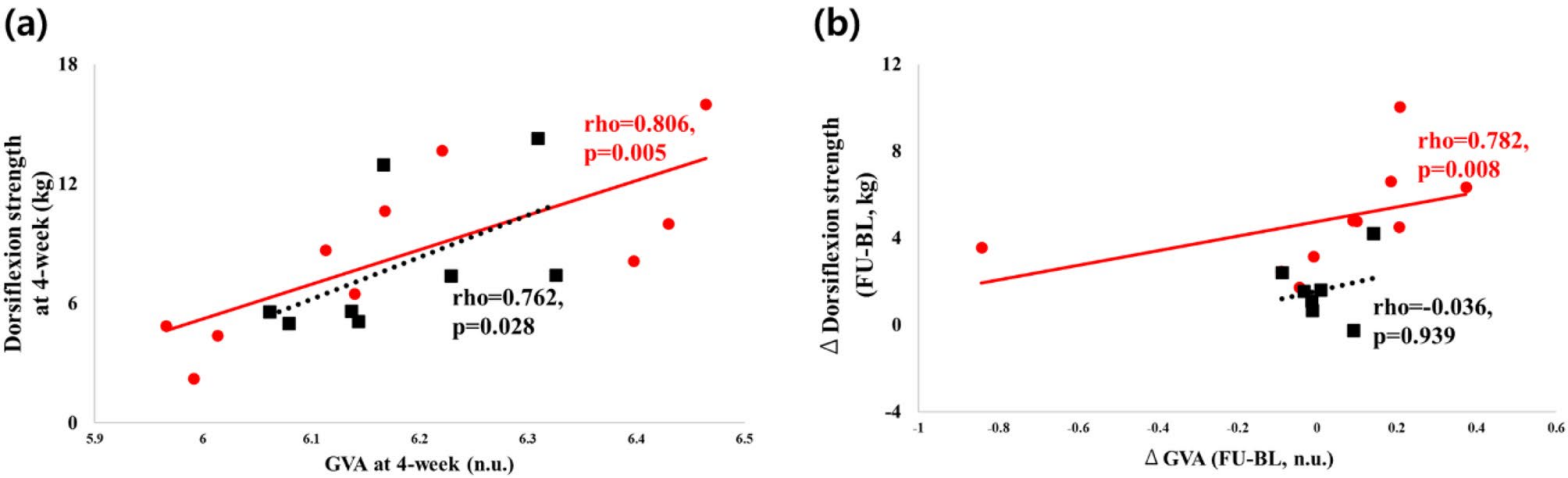
Results of the correlation analysis between gastrocnemius muscle activation and ankle dorsiflexion strength: (**a**) association between gastrocnemius muscle activation (GVA) and ankle dorsiflexion strength at 4 week assessment; and (**b**) association between improvement of gastrocnemius muscle activation and ankle dorsiflexion strength at 4 week period.

At 4 week, both groups showed a significant correlation between GVA and ankle dorsiflexion strength (IG: rho = 0.806, *p* = 0.005; CG: rho = 0.762, *p* = 0.028, Fig. 4a). However, delta correlation from baseline to 4-week (higher ∆GVA and greater ∆dorsiflexion strength) was only significant in the IG (rho = 0.782, *p* = 0.008, Fig. 4b).

There were no significant changes regarding daily activities and mobility within and between groups (Table 2). However, the IG showed a trend for improvement in the IADL score from baseline to 4 week (5.53 ± 0.68 vs. 6.2 ± 0.53, *p* = 0.058).

**Table 2:** Daily activity and mobility assessments.

	Intervention (IG) (n = 10)		*p* value: BL vs. 4W (Effect-size)	Control (CG) (n = 8)		*p* value: BL vs. 4W (Effect-size)	*p* value: IG vs. CG at 4W (Effect-size)	*p* value: group* assessment (Effect-size)
	Baseline (BL)	4 week (4W)		Baseline (BL)	4 week (4W)			
Katz index of independence in ADL (score)	5.27 ± 0.51	5.54 ± 0.29	0.285 (0.217)	5.88 ± 0.12	6.00 ± 0.00	0.285 (0.535)	0.104 (0.705)	0.633 (0.227)
Lawton-Brody IADL (score)	5.53 ± 0.68	6.20 ± 0.53	0.058 (0.366)	7.00 ± 0.43	7.13 ± 0.54	0.555 (0.101)	0.221 (0.611)	0.186 (0.655)
Mobility/ tiredness (score)	4.50 ± 0.29	4.40 ± 0.29	0.651 (0.115)	3.25 ± 0.72	3.63 ± 0.75	0.734 (0.195)	0.334 (0.525)	0.673 (0.200)

Variables are expressed as means ± standard errors. ADL: Activities of Daily Living; IADL: Instrumental activities of daily living.

## Discussion

This secondary analysis of our initial study^21^ demonstrated that daily 4-week, home-based, self-administered E-Stim to the gastrocnemius muscle was able to increase ankle dorsiflexion strength in individuals with musculoskeletal PASC. In addition, the magnitude of increase in ankle dorsiflexion strength was positively correlated with gastrocnemius muscle activation. However, these findings did not elicit changes in daily activities of PASC patients.

The present study demonstrated that only those PASC patients who underwent active E-Stim to the gastrocnemius muscle for 4 weeks had a significant effect in ankle dorsiflexion strength. One could think that dorsiflexion strength would be enhanced by stimulating only its effector muscle (i.e., anterior tibialis). However, isometric co-contraction of the antagonist muscle via E-Stim can also contribute to strength improvement^39,40^. For instance, studies have shown that ankle dorsiflexion strength can be more potentialized by the stimulation of the antagonist muscle such as the tricep surae^39,40^, since it reflects the propulsion power generation of ankle movement^41^. While stimulating both slow and fast motor units in this muscle group, coordination^42^ and function can be improved, leading to greater ankle strength^43^. Although only the IG showed a trending improvement for instrumental activities of daily living (IADL) from baseline to 4 weeks (*p* = 0.058), we believe that a 4-week time frame was not sufficient to demonstrate significant changes in IADL and mobility characteristics. This has been echoed by a recent study in moderate to severely affected COVID-19 patients who reported improvement in daily activities not sooner than 8 weeks of rehabilitation^44^. Noteworthy, the very large effect size (d = 1.2) of this outcome shows potential for longer explorations.

In addition, the present study revealed a significant correlation between ankle dorsiflexion strength and gastrocnemius muscle activation at the endpoint assessment in both groups (Fig. 3a). This supports the hypothesis for a possible association between ankle dorsiflexion and its antagonist muscle effector, validating the portable dynamometer as a reliable tool to evaluate activity of antagonist muscles during strength tests^30^. However, when exploring the magnitude of improvement between gastrocnemius activation and ankle dorsiflexion strength at 4 weeks, only the IG reported a positive correlation (Fig. 3b). Perhaps the daily gastrocnemius activation via E-Stim in this cohort may have aided in the improvement of ankle strength as this antagonist muscle’s crucial role is to provide stability to the feet by allowing forward movement of the tibia in relation to the talus^45^. However, it is difficult to confirm if these findings were related to stability improvement or even via neural effect. Therefore, these hypotheses must be confirmed via gait, balance, or nerve conduction objective assessments.

Several studies have reported E-Stim therapy improves muscle function via simple muscle fiber activation^13,46^. Particularly, this modality aligns for patients with PASC as this population should avoid vigorous exercise due to their known silent hypoxia and exercise intolerance^12^. Hence, E-Stim has shown effectiveness in improving strength without lowering oxygen saturation levels, despite providing less muscle contraction intensity than traditional exercise^21^.

The current used in the present study (HVPAC) elicits electrical pulses at low and high frequencies (20–121 Hz). This intermittent mode delivers neuromuscular benefits in different manners. In one side, high frequency (> 80 Hz) can generate greater muscle contractions, maximize muscle activation, and minimize fatigue^47^. On the other hand, low frequencies (30–50 Hz) favor the activation of motor axons^48^. The intensity of E-Stim also plays a major role for achieving muscle activation without causing discomfort or injury. In healthy subjects, Vromans & Faghri et al.^47^demonstrated that intensity is inversely proportional to frequency, thus, low intensity currents can elicit significant muscle contraction at high frequencies^47^. Under this concept, the present study’s low intensity current (< 1 mA)^49^ combined with intermittent low-to-high frequencies was able to elicit muscle contraction and relaxation without reaching tetanic contraction as confirmed via sEMG (Fig. 1E). As demonstrated in the initial results of this study, this safe current enhanced muscle oxygen recovery as well^21^. Therefore, this modality could potentially facilitate a gradual introduction to exercise and physical activity in the PASC population, enhancing muscle properties, circumventing pronounced fatigue and oxygen consumption, and deteriorated respiratory exchange ratios typically provoked by voluntary contractions^42^. Nonetheless, exercise monitored protocols are needed to confirm this statement.

In the traditional healthcare paradigm, access to certain treatments and interventions is often contingent upon geographic location, socioeconomic status, and availability of healthcare professionals for supervised interventions^50^. An individual’s ability to self-administer therapy through E-Stim treatments could therefore also potentially help improve health equity^50,51^. The present study’s therapy modality dramatically alters this landscape^51^ and provide a pathway for individuals who may not have easy access to clinical settings or specialized healthcare providers to still receive effective therapy. This treatment modality could be particularly beneficial for patients living in remote areas, individuals with mobility limitations, or those who are economically disadvantaged and cannot afford frequent clinic visits. Such an approach could also empower patients by actively involving them in their own healthcare, potentially increasing their satisfaction with the treatment. In essence, the findings of this study open the doors for a more inclusive health intervention strategy, where the benefits of treatments like E-Stim are not restricted by socioeconomic or geographic limitations, thus contributing to greater health equity. Future research should aim to further validate this approach in larger and more diverse populations, potentially tailoring it to individual needs, to capitalize on its potential to democratize access to effective healthcare interventions.

## Limitations

This study has several limitations. The sample size is relatively small, thus, potential confounders such as BMI was not adjusted to the main analyses. Nonetheless, this adjustment was included in the supplementary material showing similar results to the main analyses. Additional functional assessments (e.g., gait and balance tasks, six-minute walking tasks, or other functional measurement) are required to meaningfully indicate the effect of E-Stim therapy in physical performance of the PASC population. Other factors that may influence daily activities in our cohort were not considered (e.g., work status, family life, previous diagnosis of mental illness or physical illness, etc.). The number of muscle twitches along with their amplitude evoked by this HVPAC was not presented. It is difficult to ascertain if each electrical pulse or pulse packet (5 pulses) is equal to an individual muscle twitch. An additional sEMG analysis is needed, including various individual factors per patient to calculate the number of muscle twitches (contractions) with their respective amplitude. The reason is that every patient will elicit different data depending on such factors. Then a statistical method, including all patients’ data is required to calculate an average of such contractions and amplitude. It is important to note that the scope of this manuscript is clinical, thus, we have described the output signal from the device (per the user’s manual and sponsor consultation) and the effect (input signal) on the muscle tissue (per sEMG). Another manuscript is warranted to describe these technical calculations. Moreover, three patients in the CG recognized they had a sham device during the study, however they were not unblinded. There was no objective assessment of adherence to this protocol, nor method to confirm compliance to E-Stim therapy but verbally. Furthermore, the dynamometer assessment of this study has limitations in assessing changes in muscle strength over time or 'Force–time curve'. In addition, the modality to assess plantar flexion via this dynamometer requires placing a band strap around the subject’s hip^29^; thus, this method was excluded to avoid patient burden. Lastly, the anterior tibialis muscle was not stimulated for safety reasons, its thin composition is propense for fatigue when receiving long E-Stim periods. The proximity of the anterior tibialis to the tibia represents risk for electrode pads misplacement in untrained users, leading to pain or skin irritation.

## Conclusion

Daily self-administered gastrocnemius E-Stim over a 4 week period resulted in enhanced ankle dorsiflexion strength in individuals experiencing persistent LE musculoskeletal deconditioning due to PASC. These findings were associated with improvement of gastrocnemius muscle activation. However, this short-term therapeutic intervention did not elicit changes in activities of daily living, thus additional research involving larger sample sizes and longer interventions is warranted to observe changes in physical and daily activity outcomes. In addition, future studies should consider using more precise equipment, such as an isokinetic dynamometer, to accurately measure the improvement in muscle strength following interventions. Future studies are also advised to delve into the association between improved dorsiflexion ankle strength and gait, balance, nerve conduction, and daily physical activities.

## Data Availability

The data that support the findings of this study are not publicly available but are available from the corresponding author BN, bijan.najafi@bcm.edu upon reasonable request.

## References

[1] Agergaard, J. *et al.* Myopathic changes in patients with long-term fatigue after COVID-19. *Clin. Neurophysiol.* **132**, 1974–1981 (2021). 34020890 10.1016/j.clinph.2021.04.009 PMC8102077

[2] Paneroni, M. *et al.* Muscle strength and physical performance in patients without previous disabilities recovering from COVID-19 pneumonia. *Am. J. Phys. Med. Rehabil.* **100**, 105–109 (2021). 33181531 10.1097/PHM.0000000000001641

[3] Alkodaymi, M. S. *et al.* Prevalence of post-acute COVID-19 syndrome symptoms at different follow-up periods: A systematic review and meta-analysis. *Clin. Microbiol. Infection* **28**, 657 (2022). 10.1016/j.cmi.2022.01.014 PMC8812092 35124265

[4] Zulbaran-Rojas, A. *et al.* Safety and efficacy of electrical stimulation for lower-extremity muscle weakness in intensive care unit 2019 Novel Coronavirus patients: A phase I double-blinded randomized controlled trial. *Front. Med.* **9**, 1017371 (2022). 10.3389/fmed.2022.1017371 PMC9763311 36561714

[5] Akbarialiabad, H. *et al.* Long COVID, a comprehensive systematic scoping review. *Infection* **49**, 1163–1186 (2021). 34319569 10.1007/s15010-021-01666-x PMC8317481

[6] Ramírez-Vélez, R. *et al.* Reduced muscle strength in patients with long-COVID-19 syndrome is mediated by limb muscle mass. *J. Appl. Physiol.* **134**, 50–58 (2023). 36448687 10.1152/japplphysiol.00599.2022 PMC9762963

[7] Piotrowicz, K., Gąsowski, J., Michel, J.-P. & Veronese, N. Post-COVID-19 acute sarcopenia: physiopathology and management. *Aging Clin. Exp. Res.* **33**, 2887–2898 (2021). 34328636 10.1007/s40520-021-01942-8 PMC8323089

[8] Shanbehzadeh, S., Tavahomi, M., Zanjari, N., Ebrahimi-Takamjani, I. & Amiri-Arimi, S. Physical and mental health complications post-COVID-19: Scoping review. *J. Psychosomatic Res.* **147**, 110525 (2021). 10.1016/j.jpsychores.2021.110525 PMC8133797 34051516

[9] Silva, C. C. *et al.* Muscle dysfunction in the long coronavirus disease 2019 syndrome: Pathogenesis and clinical approach. *Revi. Med. Virol.* **32**, e2355 (2022). 10.1002/rmv.2355 PMC9111061 35416359

[10] Arena, R. *et al.* An evolving approach to assessing cardiorespiratory fitness, muscle function and bone and joint health in the COVID-19 Era. *Curr. Problems Cardiol.* **47**, 100879 (2022). 10.1016/j.cpcardiol.2021.100879 PMC8093163 34103194

[11] Connolly, B. *et al.* Exercise rehabilitation following intensive care unit discharge for recovery from critical illness. *Cochrane Datab. Syst. Rev.*10.1002/14651858.CD008632.pub2 (2015). 10.1002/14651858.CD008632.pub2 PMC6517154 26098746

[12] Singh, I. *et al.* Persistent exertional intolerance after COVID-19: Insights from invasive cardiopulmonary exercise testing. *Chest* **161**, 54–63. 10.1016/j.chest.2021.08.010 (2022). 34389297 10.1016/j.chest.2021.08.010 PMC8354807

[13] Šarabon, N., Kozinc, Ž, Löfler, S. & Hofer, C. Resistance exercise, electrical muscle stimulation, and whole-body vibration in older adults: Systematic review and meta-analysis of randomized controlled trials. *J. Clin. Med.* **9**, 2902 (2020). 32911822 10.3390/jcm9092902 PMC7563530

[14] Fukagawa, N. K., Brown, M., Sinacore, D. R. & Host, H. H. The relationship of strength to function in the older adult. *J. Gerontol. Series A Biol. Sci. Med. Sci.* **50**, 55–59 (1995). 10.1093/gerona/50A.Special_Issue.55 7493219

[15] Landers, K. A., Hunter, G. R., Wetzstein, C. J., Bamman, M. M. & Weinsier, R. L. The interrelationship among muscle mass, strength, and the ability to perform physical tasks of daily living in younger and older women. *J. Gerontol. Series A Biol. Sci. Med. Sci.* **56**, B443–B448 (2001). 10.1093/gerona/56.10.B443 11584029

[16] Edemekong, P. F., Bomgaars, D., Sukumaran, S. & Levy, S. B. Activities of daily living. (2019). 29261878

[17] Cojocaru, D.-C., Postolache, P., Petrariu, F. & Negru, R. Correlations between IADL scale and clinical parameters in severe-to-very severe COPD patients. *Med. Surg. J.* **123**, 413–418 (2019).

[18] Pizarro-Pennarolli, C. *et al.* Assessment of activities of daily living in patients post COVID-19: A systematic review. *PeerJ* **9**, e11026 (2021). 33868804 10.7717/peerj.11026 PMC8034364

[19] Karatzanos, E. *et al.* Electrical muscle stimulation: an effective form of exercise and early mobilization to preserve muscle strength in critically ill patients. *Crit. Care Res. Practice*10.1155/2012/432752 (2012). 10.1155/2012/432752 PMC3321528 22545212

[20] Falavigna, L. F. *et al.* Effects of electrical muscle stimulation early in the quadriceps and tibialis anterior muscle of critically ill patients. *Physiotherapy Theory Practice* **30**, 223–228 (2014). 24377663 10.3109/09593985.2013.869773

[21] Zulbaran-Rojas, A. *et al.* Electrical stimulation to regain lower extremity muscle perfusion and endurance in patients with post-acute sequelae of SARS CoV-2: A randomized controlled trial. *Physiol. Rep.* **11**, e15636 (2023). 36905161 10.14814/phy2.15636 PMC10006649

[22] Schriwer, E., Juthberg, R., Flodin, J. & Ackermann, P. W. Motor point heatmap of the calf. *J. Neuro Eng. Rehabil.* **20**, 28 (2023). 10.1186/s12984-023-01152-5 PMC9976413 36859293

[23] Merry, K., Napier, C., Waugh, C. M. & Scott, A. Foundational principles and adaptation of the healthy and pathological achilles tendon in response to resistance exercise: A narrative review and clinical implications. *J. Clin. Med.* **11**, 4722 (2022). 36012960 10.3390/jcm11164722 PMC9410084

[24] Krause, D. A., Cloud, B. A., Forster, L. A., Schrank, J. A. & Hollman, J. H. Measurement of ankle dorsiflexion: a comparison of active and passive techniques in multiple positions. *J. Sport Rehabil.* **20**, 333 (2011). 21828385 10.1123/jsr.20.3.333

[25] Senergy Medical Group, L. (manufactured by Avazzia Inc., DAL, TX, US, 2017).

[26] Zulbaran-Rojas, A., Park, C., Lepow, B. & Najafi, B. Effectiveness of lower-extremity electrical stimulation to improve skin perfusion. *J. Am. Podiatr. Med. Assoc.*10.7547/20-172 (2021). 33656524 10.7547/20-172

[27] Hermens, H. J., Freriks, B., Disselhorst-Klug, C. & Rau, G. Development of recommendations for SEMG sensors and sensor placement procedures. *J. Electromyograp. Kinesiol.* **10**, 361–374 (2000). 10.1016/S1050-6411(00)00027-4 11018445

[28] Silva, P. E. *et al.* Safety and feasibility of a neuromuscular electrical stimulation chronaxie-based protocol in critical ill patients: A prospective observational study. *J. Crit. Care* **37**, 141–148. 10.1016/j.jcrc.2016.09.012 (2017). 27732921 10.1016/j.jcrc.2016.09.012

[29] Romero-Franco, N., Jiménez-Reyes, P. & Montaño-Munuera, J. A. Validity and reliability of a low-cost digital dynamometer for measuring isometric strength of lower limb. *J. Sports Sci.* **35**, 2179–2184 (2017). 27882825 10.1080/02640414.2016.1260152

[30] Billot, M., Simoneau, E., Ballay, Y., Van Hoecke, J. & Martin, A. How the ankle joint angle alters the antagonist and agonist torques during maximal efforts in dorsi-and plantar flexion. *Scandinavian J. Med. Sci. Sports* **21**, e273–e281 (2011). 10.1111/j.1600-0838.2010.01278.x 21392122

[31] Konrad, P. The ABC of EMG: A practical introduction to kinesiological electromyography. (2005).

[32] Truong Quang Dang, K., Le Minh, H., Nguyen, T. H. & Van Vo, T. Analyzing surface EMG signals to determine relationship between jaw imbalance and arm strength loss. *Biomed. Eng. Online* **11**, 1–14 (2012). 22913755 10.1186/1475-925X-11-55 PMC3494581

[33] Morris, A. D., Kemp, G. J., Lees, A. & Frostick, S. P. A study of the reproducibility of three different normalisation methods in intramuscular dual fine wire electromyography of the shoulder. *J. Electromyograp. Kinesiol.* **8**, 317–322 (1998). 10.1016/S1050-6411(98)00002-9 9785252

[34] Sleivert, G. G. & Wenger, H. A. Reliability of measuring isometric and isokinetic peak torque, rate of torque development, integrated electromyography, and tibial nerve conduction velocity. *Arch. Phys. Med. Rehabil.* **75**, 1315–1321 (1994). 7993170 10.1016/0003-9993(94)90279-8

[35] Wallace, M. & Shelkey, M. Katz index of independence in activities of daily living (ADL). *Urol. Nurs.* **27**, 93–94 (2007). 17390935

[36] Lawton, M. P. & Brody, E. M. Assessment of older people: self-maintaining and instrumental activities of daily living. *The Gerontologist* **9**, 179–186 (1969). 5349366 10.1093/geront/9.3_Part_1.179

[37] Fieo, R. A., Mortensen, E. L., Rantanen, T. & Avlund, K. Improving a measure of mobility-related fatigue (the Mobility-Tiredness Scale) by establishing item intensity. *J. Am. Geriatrics Soc.* **61**, 429–433 (2013). 10.1111/jgs.12122 PMC4047030 23452001

[38] Cohen, J. *Statistical power analysis for the behavioral sciences* (Routledge, 2013).

[39] Lake, D. A. Neuromuscular electrical stimulation: An overview and its application in the treatment of sports injuries. *Sports Med.* **13**, 320–336 (1992). 1565927 10.2165/00007256-199213050-00003

[40] Duignan, C. *et al.* in *2019 41st Annual International Conference of the IEEE Engineering in Medicine and Biology Society (EMBC).* 3803–3806 (IEEE). 10.1109/EMBC.2019.8857258 31946702

[41] Zelik, K. E. & Adamczyk, P. G. A unified perspective on ankle push-off in human walking. *J. Exp. Biol.* **219**, 3676–3683 (2016). 27903626 10.1242/jeb.140376 PMC5201006

[42] Dehail, P., Duclos, C. & Barat, M. in *Annales de réadaptation et de médecine physique.* 441–451 (Elsevier). 10.1016/j.annrmp.2008.05.001 18602713

[43] André, H.-I. *et al.* Can the calf-raise senior test predict functional fitness in elderly people? A validation study using electromyography, kinematics and strength tests. *Phys. Therapy Sport* **32**, 252–259 (2018). 10.1016/j.ptsp.2018.05.012 29883924

[44] Postolache, P. A. *et al.* Clinical features and paraclinical findings in patients with SARS CoV-2 pneumonia and the impact of pulmonary rehabilitation on the instrumental activities of daily living in POST-COVID-19 patients. *J. Personalized Med.* **13**, 182 (2023). 10.3390/jpm13020182 PMC9964772 36836416

[45] Choi, Y., Lee, S., Kim, M. & Chang, W. in *Healthcare.* 777 (MDPI).

[46] Jones, S. *et al.* Neuromuscular electrical stimulation for muscle weakness in adults with advanced disease. *Cochrane Datab. Syst. Rev.*10.1002/14651858.CD009419.pub3 (2016). 10.1002/14651858.CD009419.pub3 PMC6464134 27748503

[47] Vromans, M. & Faghri, P. Electrical stimulation frequency and skeletal muscle characteristics: effects on force and fatigue. *Eur. J. Trans. Myol.* **27**, 6816 (2017). 10.4081/ejtm.2017.6816 PMC5745385 29299218

[48] Blazevich, A. J., Collins, D. F., Millet, G. Y., Vaz, M. A. & Maffiuletti, N. A. Enhancing adaptations to neuromuscular electrical stimulation training interventions. *Exercise Sport Sci. Rev.* **49**, 244–252 (2021). 10.1249/JES.0000000000000264 PMC8460078 34107505

[49] Balakatounis, K. C. & Angoules, A. G. Low-intensity electrical stimulation in wound healing: review of the efficacy of externally applied currents resembling the current of injury. *Eplasty* **8** (2008). PMC2396465 18552975

[50] Williams, J. S., Walker, R. J. & Egede, L. E. Achieving equity in an evolving healthcare system: opportunities and challenges. *Am. J. Med. Sci.* **351**, 33–43 (2016). 26802756 10.1016/j.amjms.2015.10.012 PMC4724388

[51] Narasimhan, M., Allotey, P. & Hardon, A. Self care interventions to advance health and wellbeing: A conceptual framework to inform normative guidance. *Bmj*10.1136/bmj.l688 (2019). 30936087 10.1136/bmj.l688 PMC6441866

